# Surfactant protein D: a useful marker for differentiation of drug-induced pneumonia and bacterial pneumonia

**DOI:** 10.1186/s41479-021-00087-6

**Published:** 2021-06-05

**Authors:** Yuko Waseda, Masahide Yasui, Kousuke Kurokawa, Ryo Chikazawa, Toshihiro Takeda, Miho Mitsui, Tomoaki Sonoda, Makiko Yamaguchi, Satoshi Watanabe, Hazuki Takato, Yukari Ichikawa, Yukihiro Umeda, Masaki Anzai, Hiroshi Ueda, Kazuo Kasahara, Tamotsu Ishizuka

**Affiliations:** 1 Third Department of Internal Medicine, Faculty of Medical Sciences, University of Fukui, 23-3 Matsuoka Shimoaizuki, 910-1193 Eiheiji, Fukui Japan; 2 Department of Respiratory Medicine, Kanazawa University Graduate School of Medical Science, Kanazawa, Japan; 3 Department of Surgery, Houju Memorial Hospital, Nomi, Japan; 4 Department of Respiratory Medicine, National Hospital Organisation Nanao National Hospital, Gifu, Japan; 5 Department of Respiratory Medicine, Japan Community Health Care Organisation Kanazawa Hospital, Kanazawa, Japan; 6 Department of Respiratory Medicine, Kanazawa Municipal Hospital, Kanazawa, Japan

**Keywords:** Surfactant protein D, Drug-induced pneumonia, Bacterial pneumonia, Krebs von den Lungen-6, Surfactant protein A

## Abstract

**Background:**

Drug-induced pneumonia (d-pneumonia) and bacterial pneumonia (b-pneumonia) are often difficult to differentiate; therefore, this study examined the possibility of differentiating them using serum biomarkers.

**Methods:**

The study included 22 and 16 patients diagnosed with b- and d-pneumonia, respectively, at our institution or affiliated institutions. For d-pneumonia, the causative drug was minocycline hydrochloride in four patients, gefitinib in two patients, nivolumab in two patients, pembrolizumab in two patients, sulfasalazine in two patients, loxoprofen in one patient, Bouiougitou in one patient, edoxaban tosilate hydrate in one patient, and abemaciclib in one patient. White blood cell (WBC), C-reactive protein (CRP), Krebs von den Lungen-6 (KL-6), surfactant protein (SP)-D, and SP-A levels were measured in each patient and compared between the groups.

**Results:**

Significant differences were noted in the WBC and SP-D levels between the two groups (*P* < 0.05, *P* < 0.001), but not in the CRP, KL-6, or SP-A levels.

**Conclusion:**

The study results suggest that SP-D is a useful marker for differentiating b-pneumonia and d-pneumonia.

## Background

Drug-induced pneumonia (d-pneumonia) is an important side-effect that can occur with the use of any drug. The condition can sometimes become severe due to delay in treatment. Therefore, prompt diagnosis and treatment are necessary. Chest computed tomography (CT) characteristics of d-pneumonia are diverse, and this type of pneumonia is often difficult to differentiate from bacterial pneumonia (b-pneumonia). Thus, most cases of d-pneumonia are initially misdiagnosed as b-pneumonia and are treated with antibiotics. In this study, the usefulness of serum biomarkers (Krebs von den Lungen-6 [KL-6], surfactant protein [SP]-D, and SP-A) in the differentiation of d-pneumonia and b-pneumonia, was examined.

## Methods

The subjects were 16 and 23 patients with b- and d-pneumonia, respectively, whose serum KL-6, SP-D, and SP-A levels at the acute stage were examined at our institution or affiliated institutions between April 2003 and December 2020. The subjects did not have any disease that could cause elevated levels of biomarkers and were treated with immunosuppressive agents. KL-6 and SP-D were measured by chemiluminescent enzyme immunoassay (CLEIA), and SP-A was measured using enzyme immunoassay (EIA).

Chest CT was performed at the time of diagnosis in all cases, and the diagnosis was confirmed if the image showed consolidation or ground glass attenuation in one or both lungs. The d-pneumonia was established as a pathological condition that (1) targeted the lung tissue, (2) occurred after the use of a certain drug, (3) showed no improvement following the use of antibacterials, (4) improved at least initially with the discontinuation of the drug or with the use of anti-inflammatory drugs such as steroids, (5) showed abnormalities on chest CT images, and (6) was diagnosed following exclusion of infections, pulmonary oedema, and lymphangiosis carcinomatosa, which have clinical manifestations similar to those of d-pneumonia. The b-pneumonia was established as a condition with positive sputum results (including gram stain and culture) that improved with the administration of antibacterial drugs. Moreover, b-pneumonia diagnosis was free of all biases and included all community-acquired pneumonia cases diagnosed over a period of time. The b-pneumonia group did not include hospital-acquired pneumonia and ventilator-associated pneumonia. The β-D glucan and cytomegalovirus antigen were confirmed negative to exclude pneumocystis and cytomegalovirus pneumonia when the shadow had bilateral ground glass attenuation characteristics. Viral PCR was not performed for this study, but other viral pneumonias were excluded based on observation of the clinical course. The following blood tests were performed at the initial examination: WBC, CRP, KL-6, SP-D, and SP-A measurements. Statistical analyses were performed using Student’s unpaired t-test for serum KL-6, SP-D, SP-A, WBC, and CRP levels. The significance level was set at P < 0.05.

 This study was approved by the ethics committee of Kanazawa University Hospital (#1328) and the University of Fukui (#20200151).

## Results

### Patient characteristics

Patient characteristics are shown in Table 1. The age ranged from 26−83 years for the d-pneumonia group, with a mean age of 65 years. There were nine men and seven women. The causative drug was minocycline hydrochloride in four patients, gefitinib in two patients, nivolumab in two patients, pembrolizumab in two patients, sulfasalazine in two patients, loxoprofen in one patient, the traditional Chinese medicine Bouiougitou in one patient, edoxaban tosilate hydrate in one patient, and abemaciclib in one patient.

**Table 1:** Patient characteristics

	d-pneumonia (*n* = 16)			b-pneumonia (*n* = 22)		
age	65 (26−83)			67 (28−91)		
sex (male / female)	9 / 7			15 / 7		
	causative drug	minocycline hydrochloride	4	causative bacteria	Klebsiella pneumoniae	4
		gefitinib	2		PSSP	2
		nivolumab	2		MSSA	1
		pembrolizumab	2		unknown	16
		sulfasalazine	2			
		loxoprofen	1			
		*BOUIOUGITOU*	1			
		edoxaban tosilate hydrate	1			
		abemaciclib	1			
WBC (/µL), mean ± SD	8700 ± 6505			12468 ± 1739*		
CRP (mg/dL), mean ± SD	11.2 ± 10.4			13.6 ± 2.9		

*WBC* white blood cells, *CRP* C-reactive protein, *SD* standard deviation

*P < 0.05

The age range was 28−91 years in the b-pneumonia group, and the mean age was 67 years. There were 15 men and seven women. The causative bacteria were *Klebsiella pneumoniae* in four patients, penicillin-susceptible *Streptococcus pneumoniae* in two patients, methicillin-sensitive *Staphylococcus aureus* in one patient, and unknown in 15 patients.

### Serum biomarker results

**Fig. 1:**
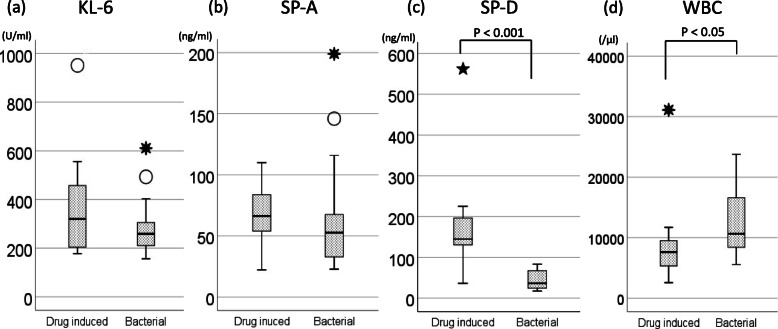
Comparison of serum biomarker and WBC levels between a drug induced group and bacterial pneumonia group. There was no significant correlation between d-pneumonia and b-pneumonia with regard to KL-6 (**a**) and SP-A (**b**) levels. However, SP-D (**c**) and WBC (**d**) levels showed a significant correlation (*P* < 0.001, *P* < 0.05). In the box plots, the boundary of the box closest to zero indicates the 25th percentile, a black line within the box marks the median, and the boundary of the box farthest from zero indicates the 75th percentile. Whiskers above and below the box indicate the 10th and 90th percentiles. Points above and below the whiskers indicate outliers outside the 10th and 90th percentiles. The dotted line indicates the cut off value. KL-6, Krebs von den Lungen-6; SP, Surfactant protein; WBC, White blood cell

There was no significant difference in KL-6 levels between the two groups. The KL-6 level was at the cut-off value (500 U/mL) or more in 4 of 16 patients (one patient was not assessed) in the d-pneumonia group and in 1 of 22 patients in the b-pneumonia group (Fig. 1a).

There was no significant difference in the SP-A levels between the two groups. The SP-A level was at the cut-off value (43.8 ng/mL) or greater in 8 of 16 patients (six patients were not assessed) in the d-pneumonitis group and in 15 of 22 patients (one patient was not assessed) in the b-pneumonia group (Fig. 1b).

The SP-D level in the d-pneumonia group was significantly elevated compared to that in the b-pneumonia group. The SP-D level was at the cut-off value (110.0 ng/mL) or greater in 11 of 16 patients (the drug was not administered in two patients) in the d-pneumonia group and in none of the 22 patients in the b-pneumonia group (Fig. 1c). A significant difference was noted in the WBC levels between the two groups (Fig. 1d), but not in the CRP levels (data not shown).

## Discussion

In this study, we compared serum KL-6, SP-D, and SP-A levels between patients with d-pneumonia and those with b-pneumonia. SP-D levels were significantly higher in the d-pneumonia group than in the b-pneumonia group. However, there was no difference in KL-6 or SP-A levels between the two groups. When d-pneumonia was determined based on the SP-D cut-off value, both sensitivity and specificity were high.

In Japan, the following clinical parameters have been used in laboratory tests for interstitial lung diseases (including IIPs): KL-6 [1], SP-A [2], and SP-D [3]. KL-6 is a high-molecular-weight glycoprotein. It is a type of MUC1 mucin recognised by a monoclonal antibody derived from mice immunised with a human lung adenocarcinoma cell line. KL-6 is produced mainly by alveolar type II epithelial cells. SP-A and SP-D are secretory glycoproteins that belong to the lung collecting family. They are similar in their molecular structure but differ in their molecular weight, molecular size, and affinity to phospholipids [4]. SP-A and SP-D are produced mainly by alveolar type II epithelial cells and have regulatory effects on innate immunity [5].

KL-6 and SP-D levels are known to be elevated in d-pneumonia [6–8]. However, one study reported that level was below the cut-off value at onset, increased over time, and then decreased again [9]. Thus, KL-6 levels might differ over time.

There are several reports regarding the differences between KL-6, SP-A, and SP-D levels. However, there have been no detailed studies. Moreover, SP-A and SP-D are secretory proteins, but KL-6 is a cell membrane protein. This difference may be a factor resulting in differences in serum concentration. Activation of a specific enzyme is necessary to cleave KL-6 from the cell membrane, and it is thought that a more severe injury must occur for the circulating KL-6 level to increase. If there is an elevation of SP-D and no elevation of KL-6, it is likely that only mild injury is present.

Both SP-A and SP-D are said to have regulatory effects on innate immunity, but there are more reports on the regulatory effects of SP-A compared to those of SP-D. SP-A is known to bind to gram*-*negative bacilli (such as *Escherichia coli*, *K. pneumoniae*, and *Haemophilus influenzae*) and gram-positive cocci (such as haemolytic streptococcus and pneumococcus). Thus, SP-A levels may be sufficiently elevated in patients with bacterial pneumonia [10, 11].

In the recent years, various drugs have been used for treating diseases such as cancer and connective tissue disorders, and the incidence of d-pneumonia has increased accordingly. Some cases of d-pneumonia improve rapidly with anti-inflammatory drugs, such as steroids or immunosuppressants, while others associated with cell damage do not improve with these drugs. The patients in this study showed relief after the use of anti-inflammatory drugs at least initially; however, d-pneumonia caused by immune checkpoint inhibitors might be exacerbated once the effect of anti-inflammatory drugs weakens, even if the subjects were relieved initially. The d-pneumonia may require early diagnosis and treatment with adequate anti-inflammatory drugs.

There have been no reports on the usefulness of SP-D as a biomarker for d-pneumonia. The results of the present study suggest that SP-D can be a useful marker to differentiate between d-pneumonia and b-pneumonia.

There were some limitations to this study. Since this was a retrospective study, there was some variability in the time until the diagnosis of d-pneumonia. The d-pneumonia caused by molecular-targeted drugs and immune checkpoint inhibitors was diagnosed relatively early; however, that caused by other drugs took longer to diagnose. In the future, blood tests should be performed at the onset of pneumonia, and prospective studies should be conducted.

## Conclusion

Serum SP-D was useful in differentiating d-pneumonia and b-pneumonia. Our study indicated that d-pneumonia can be treated early by measuring the SP-D level during initial examination.

## Data Availability

All data generated or analysed during this study are included in this published article.

## Abbreviations

b-pneumonia: Bacterial pneumonia
CLEIA: Chemiluminescent enzymeimmunoassay
CRP: C-reactive protein
CT: Computed tomography
d-pneumonia: Drug-induced pneumonia
EIA: Enzyme immunoassay
KL-6: Krebs von den Lungen-6
ROC: Receiveroperating characteristic
SD: Standard deviation
SP: Surfactantprotein
WBC: White blood cell
